# Look who’s TORking: mTOR-mediated integration of cell status and external signals during limb development and endochondral bone growth

**DOI:** 10.3389/fcell.2023.1153473

**Published:** 2023-04-19

**Authors:** Chee Ho H’ng, Ashwini Khaladkar, Alberto Rosello-Diez

**Affiliations:** ^1^ Australian Regenerative Medicine Institute, Monash University, Clayton, VIC, Australia; ^2^ Department of Biochemistry, Central University of Hyderabad, Hyderabad, India; ^3^ Department of Physiology, Development and Neuroscience, University of Cambridge, Cambridge, United Kingdom

**Keywords:** mTOR, limb development, skeletal, appendicular, cartilage, endochondral ossification

## Abstract

The balance of cell proliferation and size is key for the control of organ development and repair. Moreover, this balance has to be coordinated within tissues and between tissues to achieve robustness in the organ’s pattern and size. The tetrapod limb has been used to study these topics during development and repair, and several conserved pathways have emerged. Among them, mechanistic target of rapamycin (mTOR) signaling, despite being active in several cell types and developmental stages, is one of the least understood in limb development, perhaps because of its multiple potential roles and interactions with other pathways. In the body of this review, we have collated and integrated what is known about the role of mTOR signaling in three aspects of tetrapod limb development: 1) limb outgrowth; 2) chondrocyte differentiation after mesenchymal condensation and 3) endochondral ossification-driven longitudinal bone growth. We conclude that, given its ability to interact with the most common signaling pathways, its presence in multiple cell types, and its ability to influence cell proliferation, size and differentiation, the mTOR pathway is a critical integrator of external stimuli and internal status, coordinating developmental transitions as complex as those taking place during limb development. This suggests that the study of the signaling pathways and transcription factors involved in limb patterning, morphogenesis and growth could benefit from probing the interaction of these pathways with mTOR components.

## 1 The coordination of cell growth and proliferation during limb development

Development of the tetrapod limb requires the exquisite coordination of growth between multiple tissues (cartilage, muscle, bone, tendons, dermis, nerves, diffuse connective tissue, etc.). In most of these tissues, controlling the balance between cell proliferation and size is critical to achieve a proper size and function, and is one of the main cellular processes that are regulated by the mechanisms controlling limb development. The coupling between proliferation and cell size has been extensively studied in multiple systems, and several highly conserved pathways have been identified that link cell size to proliferative capacity. One of such pathways is the mTOR pathway (Gu et al., 2011). This review will focus on the known roles of mTOR during limb development, with a special focus on the long bones. From this review, we conclude that, besides its well-known role in controlling cell size via insulin-like growth factor (IGF) signaling, mTOR activity can crosstalk with many other pathways, such as bone morphogenetic protein (BMP), wingless/int1 (WNT), hypoxia inducible factor 1α (HIF-1α), fibroblast growth factor (FGF) and hedgehog (HH). Through these pleiotropic effects, mTOR may play important roles in limb outgrowth, chondrocyte proliferation, differentiation and metabolism, and overall in long-bone growth.

## 2 mTOR, regulators and effectors

mTOR stands for mechanistic (formerly mammalian) target of rapamycin, a macrolide produced by *Streptomyces Hygroscopicus* bacteria. Rapamycin was named after the island of Rapa Nui, where it was discovered in the early 1990 s during a genetic screen in the budding yeast, where TOR1 and TOR2 were identified as the toxic agents of rapamycin (Cafferkey et al., 1993; Kunz et al., 1993). Biochemical approaches in mammals allowed purification of mTOR and confirmed it as the target of rapamycin (Brown et al., 1994; Sabatini et al., 1994; Sabers et al., 1995).

**FIGURE 1:**
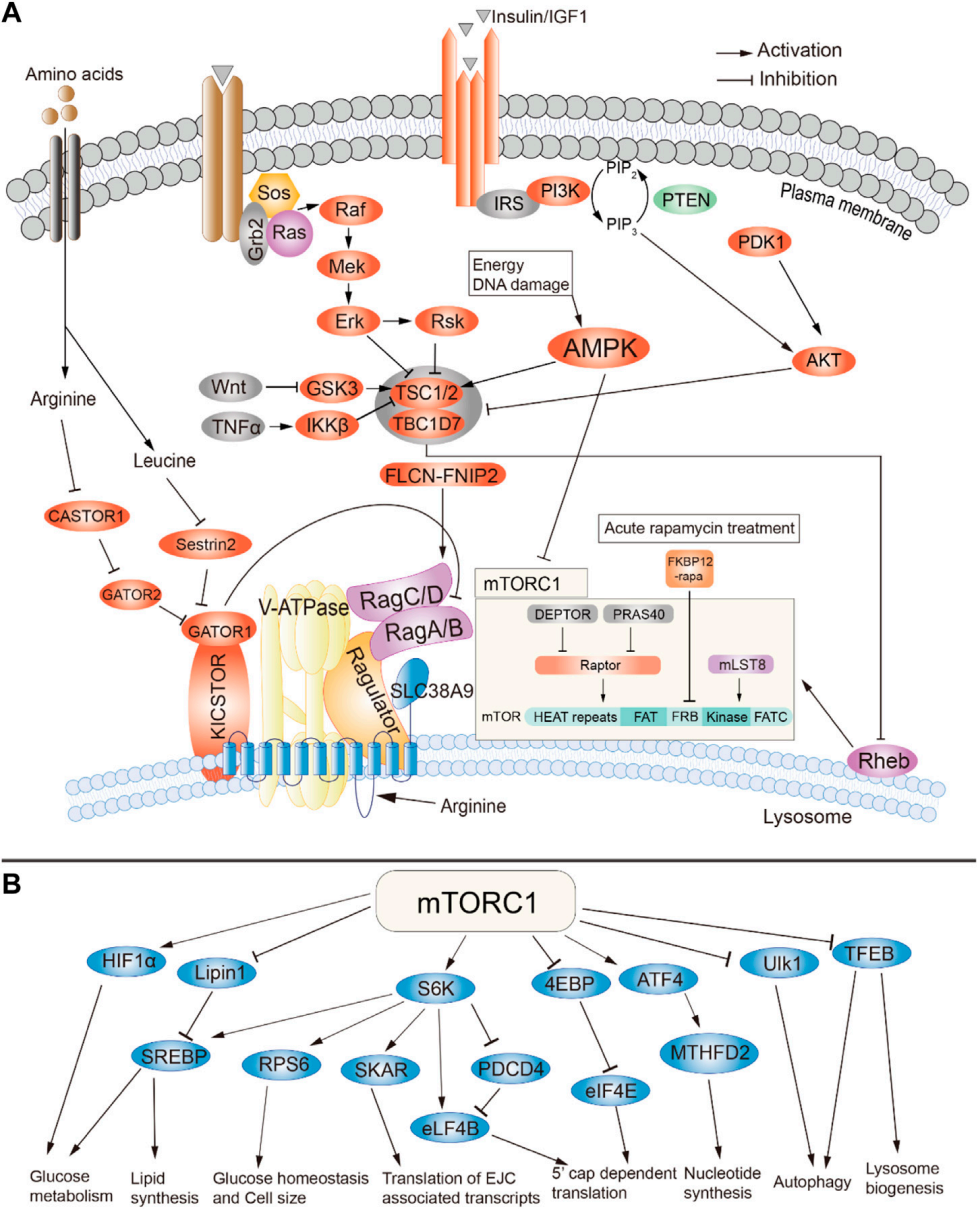
Summary of signaling through mTOR complex 1. **(A)** Activators of mTORC1. **(B)** Downstream targets and cellular effects of mTORC1. Adapted from (Wei et al., 2019), available through an CC BY license http://creativecommons.org/licenses/by/4.0/.

**FIGURE 2:**
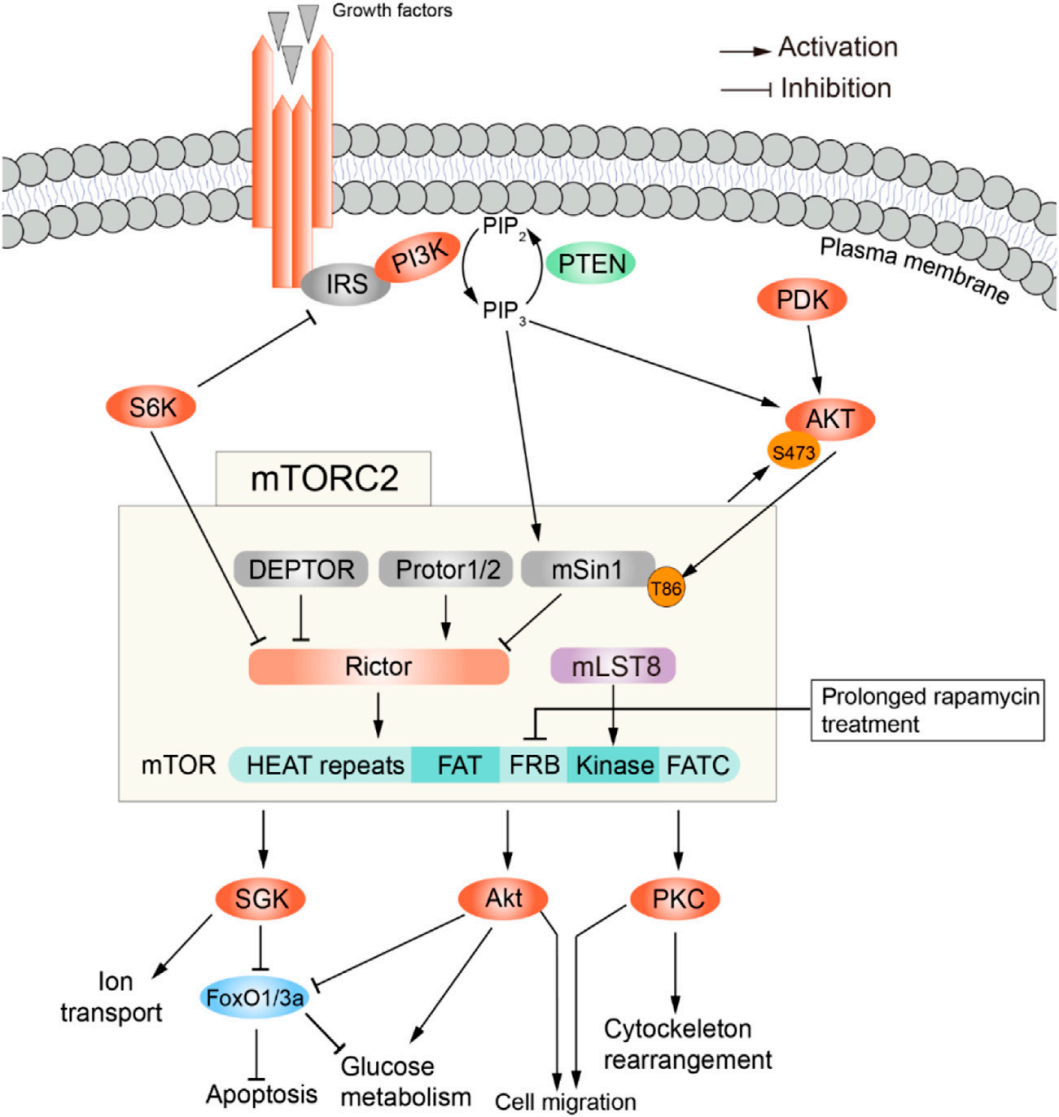
Summary of signaling through mTOR complex 2. Activators (top) and effectors (bottom) of mTORC2. Adapted from (Wei et al., 2019), available via a CC license http://creativecommons.org/licenses/by/4.0/.

mTOR is a Ser/Thr protein kinase closely associated with cell growth, survival, proliferation and tumorigenesis, especially in the abundance of amino acids, growth factors and energy. mTOR needs to form complexes with other proteins in order to exert its biological activities, named mTOR complex 1 and 2 (mTORC1 and 2, respectively). The sections below will elaborate on the known regulators and downstream effectors of both complexes (Figures 1, 2).

### 2.1 mTORC1

#### 2.1.1 Upstream signals and regulators of mTORC1

mTORC1 is composed of three core components: mTOR, Raptor, and mLST8. In addition, it contains two inhibitory subunits, PRAS40 and DEPTOR. As elaborated below, stress, growth factors, amino acid bioavailability, oxygen and other metabolites status have been identified as the upstream regulators of mTORC1 pathway (Figure 1A). At the molecular level, the activation of mTORC1 is mainly dependent on Rheb, a small GTPase that localizes to the lysosomal membrane and is negatively regulated by the tuberous sclerosis protein complex (TSC) 1 and 2 (Figure 1A) (Inoki et al., 2003; Tee et al., 2003). Growth factors, such as IGF1, can stimulate the PI3K and Ras pathways, leading to phosphorylation and thus inactivation of TSC1/2 through AKT/PKB, ERK1/2 and RSK1 kinases (Figure 1A). TSC1/2 then dissociate from the lysosomal membrane, leading to activation of mTORC1 (Inoki et al., 2002; Manning et al., 2002; Potter et al., 2002; Roux et al., 2004; Ma et al., 2005; Menon et al., 2014). This process can be further compounded by the abundance of amino acids, energy in the form of glucose and other nutrients. It has been shown that both the nutrients and the growth factors are necessary for the full activation of mTORC1 which contributes to the Rag-GTPase mediated amino acid-sensing regulation of mTORC1 (Beugnet et al., 2003; Kim et al., 2008; Sancak et al., 2008). In addition, pro-inflammatory cytokine tumor necrosis factor alpha (TNFα) also activates mTORC1 through phosphorylation-mediated inactivation of TSC1 (Lee et al., 2007). It was also shown that the canonical WNT pathway activates mTORC1 through inhibition of GSK3-β, which phosphorylates and promotes TSC2 activity (Inoki et al., 2006). This crosstalk with other signaling pathways will be further discussed in other sections.

#### 2.1.2 Downstream effectors of mTORC1

As mTORC1 is activated in the presence of nutrients, its downstream effectors are mainly associated with pathways related to protein and lipid biogenesis, cell growth and the accumulation of cell mass. mTORC1 control protein synthesis through phosphorylation of 4E-BP1 and S6K1 (Figure 1B). 4E-BP1 phosphorylation attenuates binding to eukaryotic initiation factor 4 E (eIF4E), thus allowing formation of the eIF4F complex required for protein translation. In contrast, S6K1 phosphorylation results in both increased transcription and translation. Moreover, mTORC1 also promotes protein synthesis through the regulation of TIF-1A and MAF1 (Mayer et al., 2004; Kantidakis et al., 2010; Shor et al., 2010).

mTORC1 also plays a major role in lipid synthesis, which is required for cell membrane synthesis during cell proliferation (Duvel et al., 2010; Vaidyanathan et al., 2022). mTORC1 regulates fatty acid and cholesterol synthesis via Sterol Responsive Element Binding Protein 1 and 2 (SREBP1/2). mTORC1 is required for cleavage and nuclear translocation of SREBP1/2, which leads to the expression of lipogenic genes (Porstmann et al., 2008; Duvel et al., 2010; Li et al., 2010; Wang et al., 2011). In *Drosophila*, silencing of dSREBP caused a reduction in cell and organ size and impaired the stimulation of cell growth by dPI3K (Porstmann et al., 2008). Genetic studies in mice have shown that SREBP1 preferentially regulates fatty acid biosynthesis while SREBP2 mainly controls the expression of genes related to cholesterol synthesis (Horton et al., 2003). S6K1 and/or Lipin-1 have been shown to be the mediators between mTORC1 and SREBP1/2 (Duvel et al., 2010; Li et al., 2011; Peterson et al., 2011; Wang et al., 2011). Moreover, mTORC1 also promotes expression of the master regulator of adipogenesis, PPAR-γ (Kim and Chen, 2004; Zhang et al., 2009).

### 2.2 mTORC2

#### 2.2.1 Upstream regulators of mTORC2

mTORC2 is comprised of mTOR, DEPTOR, PRAS40, mLST8, mSin1, Protor1/2, and Rictor, with DEPTOR being the only inhibitory subunit. In addition, rapamycin treatment affects mTORC1 and mTORC2 differently: acute treatment suffices to inhibit mTORC1, while mTORC2 requires prolonged treatment to get inhibited (Sarbassov et al., 2006). As shown in Figure 2, growth factors (Insulin/IGF) are the main activators of this complex. In fact, mTORC2 was identified in 2004 as the elusive ‘second’ insulin-responsive AKT kinase. The most accepted model is as follows: PI3K, activated by growth factor-receptor signaling, phosphorylates the plasma membrane phospholipid PIP2 to generate PIP3 (phosphatidylinositol 3,4,5-trisphosphate); PIP3 interacts with the pleckstrin homology (PH) domains within AKT and PDK1, triggering their translocation from the cytosol to the plasma membrane; there, PDK1 phosphorylates AKT in Thr308, partially activating it. This is sufficient to catalyze Sin1 phosphorylation within mTORC2, which then phosphorylates AKT at Ser473 to fully activate it. However, this model was challenged by a study reporting that phosphorylation of Sin1 impairs mTORC2 activity in mouse embryonic fibroblasts (Liu et al., 2013). There are also growth factor-independent activation mechanisms (Figure 2). For example, in specific cell types mTORC2 is involved in mechanically induced activation of *β*-catenin, such that inhibition of Rictor disrupts mechanically induced cytoskeletal reorganization (Lewis et al., 2020). Moreover, mTORC2 can be activated by association with ribosomes (Oh et al., 2010; Zinzalla et al., 2011). Lastly, p300-mediated acetylation of Rictor seems to potentiate IGF-1-induced mTORC2 kinase activity at Ser473 of AKT (Glidden et al., 2012), and is in general a positive regulator of mTORC2 activity (Singh et al., 2016). Intriguingly, mTORC2 location inside the cell shows broad heterogeneity depending on how it is activated. Indeed, it has been found at the plasma membrane, mitochondria, and sub-endosomal vesicles, by tracing the localization of mSin1 (Ebner et al., 2017). In summary, more work is needed to further elucidate the mechanisms of mTORC2 activation, and how this relates to its subcellular location.

#### 2.2.2 Downstream effectors of mTORC2

Once activated, mTORC2 regulates cell survival, cell migration, cytoskeletal rearrangements and glucose metabolism by mediating the phosphorylation of conserved motifs in AGC kinases (i.e., PKA, PKC, PKG). This promotes their maturation, stability and allosteric activation (Cameron et al., 2011; Baffi et al., 2021). Another likely phosphorylation target is Serum- and Glucocorticoid-induced Kinase 1 (SGK1), which in turn regulates sodium transport in kidney epithelial cells (Lang and Pearce, 2016). SGK also decreases nuclear localization of the transcription factor FOXO3a and its DNA-binding activity (Brunet et al., 2001), thus inhibiting apoptosis and promoting survival.

## 3 Role of mTOR in limb outgrowth, morphogenesis and skeletal development

**FIGURE 3:**
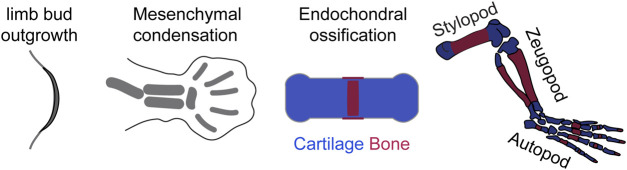
Steps in tetrapod limb development where mTOR may play a role.

For the purposes of this review, tetrapod limb development will be roughly divided into three processes (Figure 3): 1) limb outgrowth and patterning; 2) Chondrocyte differentiation after the formation of mesenchymal condensations; 3) Bone growth driven by endochondral ossification.

### 3.1 mTOR interactions with the limb outgrowth and patterning programs

**FIGURE 4:**
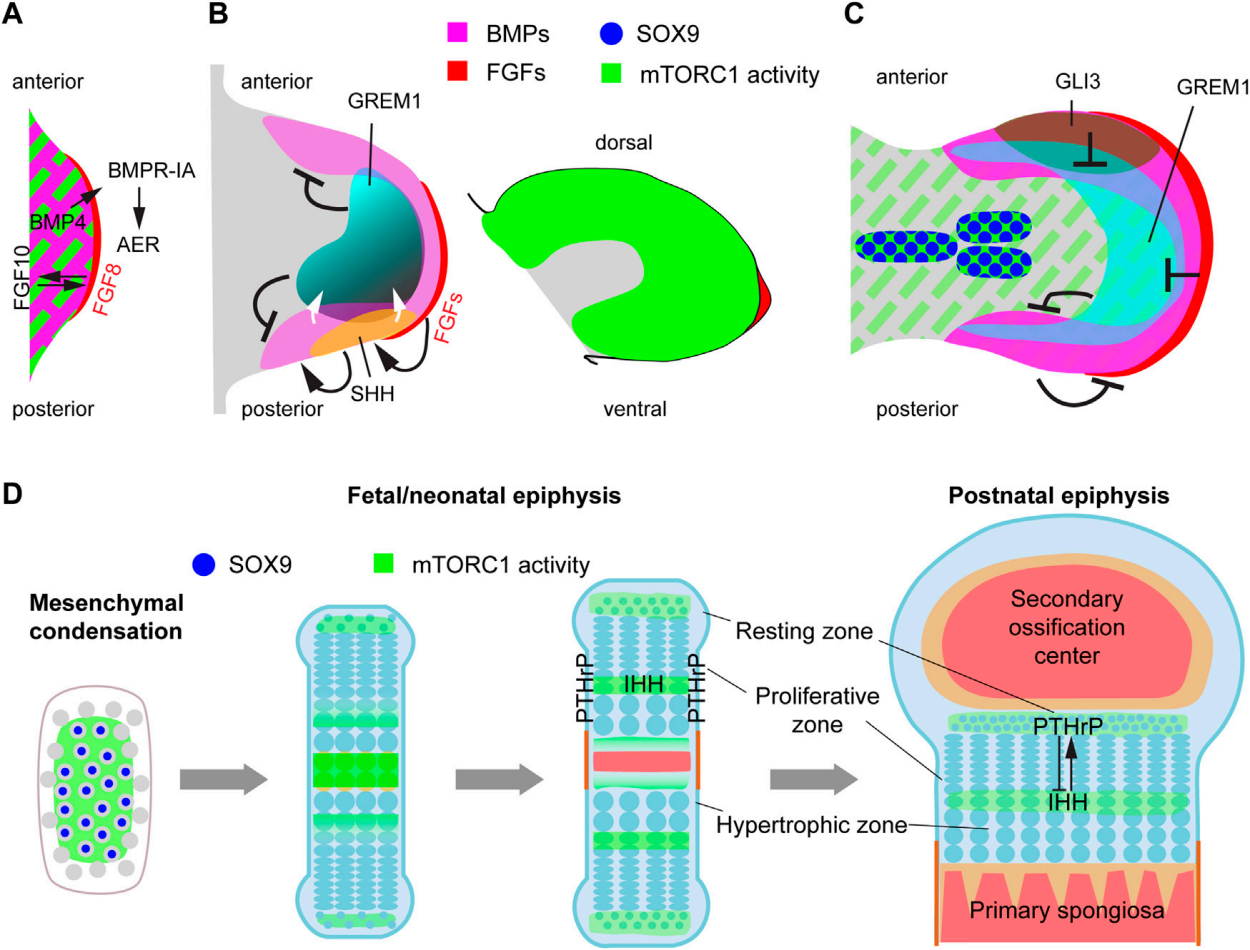
mTORC1 interacts with multiple signaling pathways and transcription factors involved in limb outgrowth, progression of skeletal condensations and bone growth. **(A)** Schematic representation of the signaling events involved in early limb-bud induction. We propose that mTORC1 could be involved in the EMT process that gives rise to the limb mesenchyme (Gros and Tabin, 2014). **(B)** During the patterning phase, mTORC1 activity is high in most of the limb mesenchyme, but seems to be only required for cell growth and timely progression of limb development (Jiang et al., 2017). **(C)** Interacting with BMP and HIF-1α pathways, mTORC1 is required for the transition of the initial condensations towards proliferative cartilage (Jiang et al., 2017; Iezaki et al., 2018; Lee et al., 2018). **(D)** Endochondral ossification process, including growth plate dynamics and main sites of mTORC1 action.

The limb primordia, or limb buds, arise as outgrowths from the lateral plate mesoderm, due to epithelial-to-mesenchymal transition of the coelomic epithelium (Gros and Tabin, 2014). The limb mesenchyme then expands in response to its interactions with the overlying epithelium (Figure 4A), A key event is the induction of an epithelial signaling center at the distal tip, the apical ectodermal ridge (AER), due to BMP4 signaling from the mesenchyme to BMP receptor IA in the epithelium (Ahn et al., 2001; Pizette et al., 2001). The AER is necessary (Saunders, 1948) and sufficient (Rosello-Diez and Torres, 2011) to promote the outgrowth of the limb. Also critical for limb outgrowth is the concomitant stabilization of *Fgf10* expression in the limb field, which then signals to the overlying AER and activates WNT3A/β-catenin signaling, in turn inducing *Fgf8* expression, which maintains *Fgf10* expression in the mesenchyme and is critical for limb outgrowth (Ahn et al., 2001; Kawakami et al., 2001; Pizette et al., 2001).

The role of mTOR in the limb outgrowth process has not been specifically addressed, as the earliest genetic conditional deletion of key pathway components such as *Raptor* were done via *Prrx1-Cre*, which is only active once the limb bud induction process is finalized (Logan et al., 2002). However, given the role of mTORC1 and 2 in mediating EMT in organogenesis and cancer (Gulhati et al., 2011; Karimi Roshan et al., 2019), and that the mTORC1 readout p-S6 is present in most of the mesenchyme of the early limb bud (Figures 4A,B), we propose that mTOR (or at least mTORC1) may play a role in the EMT process that leads to limb bud initiation. Supporting this possibility, artificial overexpression of RhoA represses mTORC1 signaling in mammalian cells (Gordon et al., 2014) and interferes with EMT-initiated limb outgrowth in chicken (Gros and Tabin, 2014).

After induction, the limb bud undergoes significant growth. Deletion of *Raptor* with *Prrx1-Cre* showed that mTORC1 signaling in the limb mesenchyme is required for the normal size of both the limb bud and its individual cells, but relatively dispensable for skeletal patterning (Jiang et al., 2017). A potential link between mTOR and limb growth is the FGF-IGF connection. It has been shown that the maintenance of limb growth by AER-FGFs requires the induction and signaling of IGF1 in the subridge mesenchyme, at least in the chicken embryo (Dealy et al., 1996). Moreover, insulin/IGF signaling in the limb mesenchyme is mediated by PI3K/AKT (Knobloch et al., 2008), which, as mentioned above, is an upstream regulator of mTORC1. Since mTORC1 activity is detected in the limb mesenchyme (Figure 4B), it is possible that mTORC1 mediates part of the effects of AER-FGFs on limb growth. Although this possibility has not been directly explored, there are studies in which one copy of *Rps6* (encoding ribosomal protein S6, a mediator of mTORC1 activity) was removed in mouse limb buds using *Prrx1-Cre,* leading to smaller limb buds by embryonic day (E) 11.5, and agenesis of the humerus, radius and most anterior digit at E17.5 (Tiu et al., 2021). It is noteworthy that the hindlimb, where *Prrx1-Cre* is activated later and more sparsely than in the forelimb (Logan et al., 2002), was mostly unaffected in this study, suggesting that *Rps6* full dosage is only needed in early limb growth.

### 3.2 mTOR role in chondrocyte differentiation after mesenchymal condensation

During the limb growth process, mesenchymal cells far from the AER start coalescing into the so-called mesenchymal condensations (Figure 3), which will give rise to the skeletal elements in a complex but stereotypical manner (Markman et al., 2023). In this differentiation process, the extracellular space between cells collapses, increasing the packing density. This requires partial replacement of the extracellular matrix (ECM) with new ECM components. Also, as density increases, cells become rounder and lose their filopodia (Thorogood and Hinchliffe, 1975). Subsequently, the condensations enlarge through cell proliferation, and a layer of elongated cells surrounds the condensation, forming the perichondrium. BMP signaling induces condensation and proliferation of mesenchymal cells and promotes their survival (Yoon et al., 2005). Moreover, these condensations become hypoxic, and the HIF-1α pathway is required for their progression towards cartilage cells or chondrocytes (Provot et al., 2007). Interestingly, levels of the mTORC1 readout p-S6 are high in the condensations, while they decrease in the rest of the mesenchyme (Figure 4C), suggesting a role in their formation/progression and potential interactions with the BMP and HIF1-α pathways. Indeed, BMPs have been shown to induce mTORC1 activation via the ALK3 receptor and Smad4-mediated inhibition of PTEN (Lim et al., 2016; Lee et al., 2018). mTORC1, in turn, is required for the translational control of SOX9, a key transcription factor in the progression towards cartilage (Iezaki et al., 2018). Moreover, mTORC1 has been shown to upregulate HIF-1α protein levels in the cartilage, which is critical for the control of glucose metabolism, proliferation and differentiation in chondrocytes (Lee et al., 2018).

### 3.3 mTOR and endochondral ossification

**FIGURE 5:**
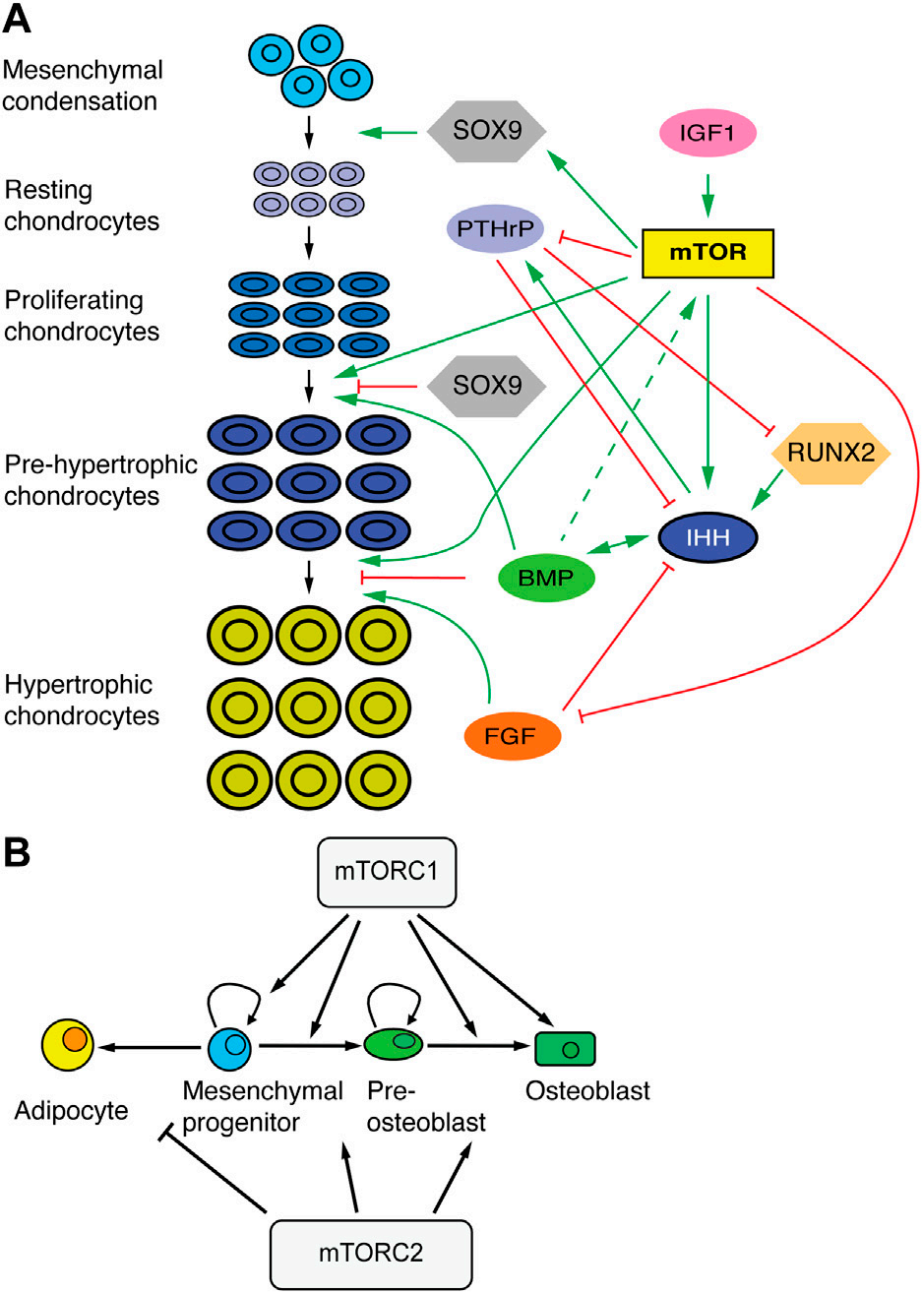
mTOR in skeletal development. **(A)** Schematic of chondrocyte transitions during cartilage growth and their regulators. mTOR plays an integrative role at the intersection of progenitor/stem cell proliferation, chondrocyte differentiation and cell size control. **(B)** Effects of mTORC1 and 2 in the differentiation of mesenchymal progenitors. Based on (Chen and Long, 2018).

The process of endochondral ossification plays a predominant role in long-bone growth. In this process, a cartilage template (formed when mesenchymal cells in the condensations transition to chondrocytes) is progressively replaced by bone. From the ends towards the center of the bone, chondrocytes undergo subsequent differentiation stages (Figures 4D, 5A). Round resting chondrocytes constitute a reserve pool that with a certain frequency gives rise to proliferative chondrocytes arrayed in columns. Proliferating chondrocytes eventually undergo hypertrophy by increasing protein synthesis and osmotic swelling (Cooper et al., 2013), while they lay down the ECM that forms the cartilage scaffold. While some hypertrophic chondrocytes die, others transdifferentiate to osteoblasts (Yang et al., 2014; Zhou et al., 2014; Park et al., 2015). Osteoblasts can also derive from precursors located in the perichondrium (Maes et al., 2010), and regardless of their origin, lay down the bone matrix, forming the primary ossification center in the middle region of the skeletal element. This divides the cartilage template into two parts (growth plates), located at both ends, where the process continues (Figure 4D). Eventually, a secondary ossification center forms within each cartilage pole (Figure 4D). Key transcription factors like SOX9, RUNX2, MEF2C, and cell signaling pathways such as WNT, BMP, FGF, indian hedgehog (IHH) and parathyroid hormone-related peptide (PTHrP) affect chondrocyte proliferation and differentiation and osteoblast differentiation, often interacting with mTORC1 and 2 (Figures 5A, B).

#### 3.3.1 Role of mTOR in chondrocyte proliferation and hypertrophic differentiation

As a first approach to studying the role of mTORC1 in endochondral ossification, rapamycin was used to treat fetal rat metatarsal cultures or pregnant rats in the last day of gestation, finding that mTORC1 inhibition impaired fetal chondrocyte differentiation and response to insulin, but not proliferation (Phornphutkul et al., 2008). Similarly, genetic deletion of either *Mtor* or *Raptor* in the mouse cartilage impaired skeletal growth through reduced matrix production, decreased chondrocyte size and delayed chondrocyte hypertrophy (Chen and Long, 2014). While these landmark studies were important first steps, given the multiple signaling interactions and roles that mTOR can participate in, as well as the multiple states and complex spatiotemporal dynamics that chondrocytes go through, it is worth considering more recent studies as well, focused on specific aspects of chondrocyte regulation by mTOR.

Due to the continuous cartilage destruction near the bone, maintenance of the cartilage regions, and hence bone growth, requires *de novo* production of cartilage at roughly the same rate. At fetal/early postnatal stages, cartilage progenitors located in the resting zone do not self-renew and have limited proliferative potential, so that when they are recruited into the proliferative pool, they deplete the pool of progenitors and give rise to short clonal columns of chondrocytes (Newton et al., 2019). Some time after formation of the secondary ossification center (SOC), however, some resting chondrocytes start to self-renew and show higher clonogenic potential, so that the pool of resting chondrocytes is not depleted as fast, and long clonal columns of chondrocytes are formed (Newton et al., 2019). Signals from the SOC (most prominently sonic hedgehog, Shh) seem to be critical for this change of behavior, as was the activation of the mTORC1 pathway in these so-called cartilage stem cells (Newton et al., 2019). Importantly, pharmacological abrogation of HH signaling reduced proliferation in the resting zone, while ectopic activation of mTORC1 via *Tsc1* deletion led to a bias towards symmetric division in the resting zone, disorganizing cartilage structure (Newton et al., 2018; Newton et al., 2019). There is no consensus about this phenotype, however, as other group reported drastically different results (Yan et al., 2016), warranting further analyses.

Besides the control of cartilage progenitors, balanced cartilage growth requires maintenance of a relatively constant height of the different regions of the growth plate. The size of the proliferative zone is controlled by a well characterized negative feedback loop between IHH and PTHrP (Lee et al., 1996; Vortkamp et al., 1996; St-Jacques et al., 1999; Minina et al., 2001). In this loop, IHH produced by pre-hypertrophic chondrocytes induces PTHrP expression in resting chondrocytes, whereas PTHrP secreted from the resting zone promotes chondrocyte proliferation and delays differentiation, including *Ihh* expression (Figures 4D, 5A). mTOR is likely involved in this feedback loop in two different ways: via mechanotransduction-dependent *Ihh* expression, and via regulation of PTHrP signaling. Regarding the former, mechanical loading is an important regulator of chondrocyte maturation, and experiments in chicken embryos showed that elimination of muscle contraction results in mTOR inhibition in the cartilaginous growth plate (Guan et al., 2014). This led to significant inhibition of chondrocyte proliferation and reduced expression of *Ihh*. Conversely, mechanical stimulation of chondrocytes *in vitro* led to mTORC1-dependent activation of *Ihh* expression (Guan et al., 2014). Moreover, one of us (AR-D) showed that *in vivo* inhibition of SHP2, an antagonist of mTORC1-mediated mechanotransduction, leads to increased *Ihh* expression (Rosello-Diez et al., 2017). Along these lines, it was recently shown in chicken and alligator embryos that limb proportions can change in response to embryo movement, an effect due to mTOR-mediated changes in chondrocyte proliferation and specific of certain growth plates (Pollard et al., 2017).

On the other hand, mTORC1 activation has been shown to reduce expression of the PTHrP receptor in articular cartilage (Zhang et al., 2017), which could potentially happen in the growth plate cartilage as well. Moreover, Yan et al. showed that S6K1, a downstream effector of mTORC1, phosphorylates and allows nuclear translocation of HH-signaling transducer GLI2, leading to transcription of *Pthlh*, encoding PTHrP (Yan et al., 2016). The mTOR/PTHrP interaction also works in reverse. Studies of skeletal dysplasia syndromes characterized by constitutive activation of PTH/PTHrP showed reduced activities of salt inducible kinase 3 (SIK3), which caused accumulation of DEPTOR, in turn inhibiting mTORC1 and 2 activity, biasing skeletal progenitor differentiation towards fat instead of bone (Csukasi et al., 2018). This new PTH/PTHrP-SIK3-mTOR axis has been recently explored further, showing that, in the presence of nutrients, DEPTOR directly interacts with PTH1R to regulate PTH/PTHrP signaling, whereas in the absence of nutrients it forms a complex with TAZ (an effector of the Hippo pathway), to prevent its translocation to the nucleus and therefore inhibit its transcriptional activity (Csukasi et al., 2022).

Another potential signaling interaction of mTORC1 is with the canonical WNT pathway. Although related to calvarial bone (not long bones), it was shown that *Wnt10b* overexpression causes enlargement of calvarial tissue and phosphorylation of S6, both of which effects were abrogated by rapamycin (Inoki et al., 2006). Similarly, mice with reduced expression of LRP1 (a WNT receptor) had smaller than normal calvarias and reduced mTORC1 signaling. The connection seems to be mediated by WNT-dependent inhibition of GSK-3β, which via its interdependence with AMPK phosphorylates TSC2, acting as an mTORC1 inhibitor (Inoki et al., 2006). In the future, it would be interesting to test this crosstalk during endochondral ossification in the long bones.

A key signal transducer that interacts with the mTOR pathway during cartilage and skeletal growth is AKT (aka protein kinase B). AKT is the collective name of three Ser/Thr protein kinases that play key roles in cellular processes such as cell proliferation, migration, apoptosis, glucose metabolism and transcription. The functions of AKT and its downstream molecular mechanisms were explored during skeletal development using transgenic mouse embryos that expressed constitutively active AKT (Rokutanda et al., 2009). AKT positively regulated ECM production, chondrocyte growth, proliferation and maturation; associated with increased phosphorylation of FoxO3a, S6K (i.e., mTORC1 signaling) and GSK-3β in the growth plates of the AKT transgenic mice. Further examination of the downstream signaling pathways by *ex vivo* culture revealed that the AKT-mTOR pathway positively regulated proliferation, maturation and ECM production (Rokutanda et al., 2009).

mTORC2 and mTORC2-mediated activation of mTORC1 are also crucial in skeletal development. It was found that genetic deletion of *Rictor* (encoding an essential component of mTORC2) in the limb mesenchyme led to a smaller skeleton in mice (Chen et al., 2015). This phenotype is caused by delayed chondrocyte hypertrophy and not changes in cell size, proliferation, apoptosis, or ECM production (Chen et al., 2015). Furthermore, RUNX2, a master regulator of skeletal development, has been shown to activate PI3K/AKT signaling via mTORC2 in breast cancer cells (Tandon et al., 2014). However, the question of whether the RUNX2/PI3K/AKT/mTORC2 axis is conserved in skeletal development remains to be examined.

#### 3.3.2 Role of mTOR in the osteoblast/osteocyte lineage

Besides its roles in chondrocytes, mTORC1 is required for the transition of pre-osteoblasts to mature osteoblasts (Figure 5B). Indeed, *Mtor* or *Raptor* deletion (and thus perturbation of mTORC1 function) in preosteoblasts (using *Osx-Cre*) or in calvarial cultures caused reduced osteogenic capacity and delayed bone formation (Chen and Long, 2015; Dai et al., 2017; Fitter et al., 2017). Mechanistically, this was caused by reduced expression of *Runx2*, a master regulator of osteogenesis (Dai et al., 2017).

Regarding mTORC2, its inactivation via *Prrx1-Cre* mediated deletion of *Rictor* in the limb mesenchyme did not affect chondrocyte proliferation, apoptosis, cell size or matrix production, but instead caused a delay in osteoblast differentiation (Figure 5B) (Chen et al., 2015). Moreover, *Rictor* deletion in late-stage osteoblasts/osteocytes with *Dmp1-Cre* hampered load-induced bone formation, causing a reduced bone mass phenotype (Lewis et al., 2020).

mTORC2 can also interact with other signaling pathways. Through its tyrosine kinase activity, mTORC2 is involved in the activation of IGF-1R/InsR signaling, which plays an essential role in osteoblasts proliferation, survival, and differentiation during skeletal growth. Indeed, a study reported that mTORC2 is recruited to the IGF-1R/InsR via SIN1 interaction with insulin receptor substrate (IRS) (Yin et al., 2016). Another study showed that IGF-activated AKT and PI3-kinase are crucial for BMP2-stimulated *Runx2* expression and therefore osteoblast differentiation, maturation, and function in mouse metatarsal bones (Mukherjee and Rotwein, 2009) (Figure 5B).

WNT signaling has been shown to play a role in both prenatal and postnatal skeletal formation, in part interacting with mTORC1 and 2. Prenatal induction of WNT7B in the osteoblast lineage (*Col1a1-Cre*) increased bone mass via increased osteoblast number during bone formation (Chen et al., 2014). Deletion of Raptor in the osteoblast lineage reduced the WNT7B-induced phenotype. In addition, induction of WNT7B postnatally in *Runx2* ^+^ cells also enhanced bone formation (Chen et al., 2014). Moreover, WNT3A signaling via LRP5, independent of *β*-catenin, has been shown to activate mTORC2-AKT downstream of RAC1 and promote a metabolic reprogramming that is critical for osteoblast differentiation (Esen et al., 2013).

Therefore, all together, it can be inferred that mTORC1 and 2, activated by IGFs, BMPs and WNTs are involved in the regulation of osteoblast activity to maintain skeletal growth.

## 4 Conclusion. mTOR and limb development: a TORrent of possibilities

In this review, we have addressed the role and regulation of the mTOR pathway during multiple steps of tetrapod limb development, with a focus on the long bones. mTOR, as an integrator of intrinsic cellular status (e.g., metabolism, stress) and extrinsic signals (e.g., growth factors, nutrients), is in a unique position to coordinate multiple inputs into a robust growth program. In fact, its widespread expression, pleiotropic effects and ability to interact with multiple signaling pathways and transcription factors suggest that it could be involved in more processes than the ones studied so far. For example, we have already mentioned that, while no study has found a clear role for mTORC1 or 2 in limb initiation, most genetic interventions have been performed once the limb bud is already present, precluding a clear conclusion. There is also the possibility of synergies. Since mTORC1 and 2 can modulate/interact with many of the signaling pathways involved in limb patterning (BMP, FGF, HH, WNT, Figures 4B, C), it is reasonable to think that heterozygous deletions of mTORC components may synergize with genetic defects impacting said pathways. This could also have implications in other fields. For example, in the context of cancer it has been recently proposed that the interaction between HH and mTORC1, happening at the level of the primary cilium, is more widespread than currently thought (Larsen and Moller, 2020). We submit that disentangling these interactions could open new avenues to understanding the control of limb size and proportions during development and evolution, and treating limb growth disorders.
